# Quality of life among elderly patients undergoing transcatheter or surgical aortic valve replacement– a model-based longitudinal data analysis

**DOI:** 10.1186/s12955-016-0512-9

**Published:** 2016-07-26

**Authors:** Klaus Kaier, Anja Gutmann, Hardy Baumbach, Constantin von zur Mühlen, Philip Hehn, Werner Vach, Friedhelm Beyersdorf, Manfred Zehender, Christoph Bode, Jochen Reinöhl

**Affiliations:** 1 Institute of Medical Biometry and Statistics, Faculty of Medicine and Medical Center – University of Freiburg, Stefan-Meier-Str. 26, D-79104 Freiburg, Germany; 2 Department of Cardiology, Heart Center Freiburg University, Freiburg, Germany; 3 Department of Cardiovascular Surgery, Robert-Bosch-Krankenhaus, Stuttgart, Germany; 4 Department of Cardiovascular Surgery, Heart Center Freiburg University, Freiburg, Germany

**Keywords:** Quality of life, EQ-5D, Transcatheter aortic valve implantation, TAVI, Transcatheter aortic valve replacement, TAVR, Aortic valve replacement, AVR

## Abstract

**Background:**

Quality of life (QoL) measurements reported in observational studies are often biased, since patients who failed to improve are more likely to be unable to respond due to death or impairment. In order to observe the development of QoL in patients close to death, we analyzed a set of monthly QoL measurements for a cohort of elderly patients treated for aortic valve stenosis (AS) with special consideration of the effect of distance to death.

**Methods:**

QoL in 169 elderly patients (age ≥ 75 years), treated either with transcatheter aortic valve replacement (TAVR; *n* = 92), surgical aortic-valve replacement (*n* = 70), or drug-based therapy (*n* = 7), was evaluated using the standardized EQ-5D questionnaire. Over a two-year period, patients were consulted using monthly telephone interviews or outpatient visits, leading to a total of 2463 time points at which QoL values, New York Heart Association (NYHA) Functional Classification and their status of assistance were assessed. Furthermore, post-procedural clinical events and complications were monitored. Linear and ordered logistic regression analyses with random intercept were carried out, taking into account overall trends and distance to death.

**Results:**

QoL measures decreased slightly over time, were temporarily impaired at month 1 after the initial episode of hospitalization and decreased substantially at the end of life with a measurable effect starting at the sixth from last follow-up (month) before death. Many clinical complications (bleeding complications, stroke, acute kidney injury) showed an impairment of QoL measurements, but the inclusion of lagged variables demonstrated medium term (three months) QoL impairments for access site bleeding only. All other complications are associated with event-related impairments that decreased dramatically at the second and third follow-up interviews (month) after event.

**Conclusions:**

Distance to death shows clear effects on QoL and should be taken into account when analyzing QoL measures in the elderly patients treated for aortic valve stenosis.

**Trial registration:**

German Clinical Trial Register Nr. DRKS00000797

## Background

The prevalence of acquired aortic valve stenosis (AS) is on the rise in the ageing populations of the developed countries [1, 2]. Functional and quality of life (QoL) impairment as well as mortality are extremely high among symptomatic patients, and the survival and QoL benefits of surgical (SAVR) and transcatheter aortic valve replacement (TAVR) have been shown previously [3–5].

A number of studies have been published in recent years on QoL among patients with aortic valve stenosis, most of which focus on the newly available TAVR procedure. Several recent reviews provide an overview of the body of evidence and its strengths and weaknesses [6–12].

Unfortunately, these reviews report a varying and often poor quality of studies [9, 10, 12] and find many (observational) studies to be subject to survivor bias [7, 8, 10]: The cohort of patients filling out QoL questionnaires at follow-up is disproportionately composed of those who benefitted from the procedure, since patients who failed to improve are more likely to be unable to respond due to death or impairment. In addition, patient co-morbidities and available follow-up time frames are highly diverse, which underlines the need for further studies with repeated QoL measurements over a longer time period.

In this study, we aim to bridge this gap of knowledge by evaluating QoL measurements among high-risk patients with severe symptomatic aortic valve stenosis taken in the course of the prospective, medical-economic TAVI Calculation of Costs Trial (TCCT). We used monthly QoL measurements over a two year period in 169 patients in order to analyze the impact of clinical complications on QoL values, taking into account differences between treatment groups, overall trends and distance to death.

## Methods

### Data collection

The TAVI Calculation of Costs Trial (TCCT), which this study is based on, was designed as a prospective observational multicenter cohort study on elderly patients with symptomatic AS receiving either SAVR, TAVR, or best medical therapy (DRUG). This study has been approved by the institutional ethics committee (Research Ethics Committee Albert-Ludwigs-Universität Freiburg, Germany ID: 52/11), and registered in the German Clinical Trial Register (ID: DRKS00000797). All patients referred to our centers between April 2011 and October 2013 were considered for inclusion into the study. Age above 75 years was deliberately chosen as an inclusion criterion. All treatment decisions were made by a study-independent “heart team” of cardiac surgeons and cardiologists according to best clinical practice [13].

A primary focus of the study was to evaluate QoL before and after procedure as well as during a 2-year follow-up period using the EQ-5D questionnaire, a standardised instrument to measure health outcome whose design emphasizes simplicity and quick completion, improving its feasibility and thereby response rates from this study’s severely health-impaired patient population [14]. EQ-5D_Index_ values were calculated using the established German calculation formula which is based on time trade off values [15]. A total of 2463 questionnaires were completed by 169 elderly patients treated with either transcatheter aortic valve replacement (TAVR, *N* = 92, of which 30 received TAVI via the transapical approach), surgical aortic valve replacement (SAVR, *N* = 70, of which 17 received additional bypass grafting and/or mitral valve replacement), or drug-based therapy (DRUG, *N* = 7), who were tracked using monthly telephone interviews. Cardiovascular and non-cardiovascular complications, prosthetic valve associated endpoints, and therapy-specific endpoints were assessed according to the revised definitions provided by the Valve Academic Research Consortium (VARC-2) [16].

### Statistical analysis

Differences between treatment groups were analyzed using the non-parametric Wilcoxon rank-sum test. Linear and ordered logistic regression analyses with a random intercept were carried out for the EQ-5D questionnaire's EQ-5D_Index_ (continuous endpoint, 0 = worst state, 1 = best state), the 5 EQ-5D dimensions (order categorical endpoint, 1 = no problems, 2 = some problems, 3 = extreme problems), patients heart function according to the New York Heart Association (NYHA) Functional Classification and the Canadian Cardiovascular Society (CCS) Angina Grading Scale. In addition, the current status of assistance at follow-up was retrieved and an order categorical variable was built with the following characteristics: 1 (no external help or intra-familial help only), 2 (assisted living and/or home care services) and 3 (short or long-term care or temporal hospitalization). Distance to death was modeled as a categorical variable for the last six months before death. With respect to EQ-5D_Index_ values during follow up, overall measurement compliance was 56.6 %, with ~85 % compliance during the first three months, ~80 % compliance during the first six months, and ~73 % compliance during the first year, but only ~40 % compliance during the second year. In addition, there are a number of missing values with respect to EQ-5D dimensions, patients heart function, their current status of assistance and clinical complications. With respect to these missing values no imputation method was applied.

All analyses were performed using Stata 14 (Stata Corp., Texas. USA).

## Results

As treatment decisions were based on clinical judgement according to patient presentation and the assessment of a “heart team” there were substantial differences between groups. SAVR patients were significantly younger (*p* < 0.01) and exhibited significantly lower EuroScore I values (*p* < 0.01) than TAVR patients. In contrast, QoL values at baseline did not differ significantly between TAVR and SAVR patients (*p* = 0.41). See Table 1 for an overview of pre- and post-procedural parameters.

**Table 1:** Baseline and post-procedural monthly parameters

Baseline and post-procedural parameters (*N* = 169)						
	At baseline (*n* = 169)					
	mean	SD				
Age (in years)	82.15	5.16				
logistic EuroSCORE	16.57	11.54				
	At baseline (*n* = 169)			During follow-up (*n* = 2294)		
	Mean	SD		Mean	SD	
HrQoL_Index_	0.78	0.23		0.77	0.25	
5 EQ-5D dimensions	No problems	Some problems	Extreme problems	No problems	Some problems	Extreme problems
Mobility	50 %	50 %	0 %	39 %	58 %	3 %
Self-Care	79 %	20 %	2 %	70 %	24 %	6 %
Usual Activities	53 %	40 %	8 %	44 %	44 %	12 %
Pain/Discomfort	51 %	49 %	8 %	33 %	62 %	5 %
Anxiety/Depression	64 %	30 %	6 %	69 %	28 %	3 %
Heart Function	Mean	SD		Mean	SD	
NYHA SCORE	2.57	0.7		1.36	1.08	
CCS SCORE	1.23	1.32		0.5	0.83	
Current status of the assistance	No external help or intra-familial help only	Assisted living and/or home care services	Short or long-term care or temporal hospitalization	No external help or intra-familial help only	Assisted living and/or home care services	Short or long-term care or temporal hospitalization
	77 %	13 %	10 %	73 %	18 %	9 %
Cardiological events among TAVI and AVR patients	In-hospital	Post-discharge				
ASB life threatening/disabling	12	0				
ASB major/minor	12	0				
NASB life threatening/disabling	6	2				
NASB major/minor	11	8				
VASC major/minor	24	3				
Stroke/TIA	4	2				
AKIN 1	16	1				
AKIN 2	6	1				
AKIN 3	10	6				
Severe cardiac dysrhythmia	48	6				
Other complications	12	3				

The EQ-5DIndex is a continuous variable between 0 and 1 (0 = worst state, 1 = best state). The 5 EQ-5D dimensions as shown in Model (2) to model (6) are categorical variables (1 = no problems, 2 = some problems, 3 = extreme problems). Canadian Cardiovascular Society (CCS) Angina Grading Scale is a categorical variable between 1 (angina only during strenuous or prolonged physical activity) and 4 (inability to perform any activity without angina). New York Heart Association (NYHA) Functional Classification is a categorical variable between 1 (cardiac disease, but no symptoms and no limitation in ordinary physical activity) and 4 (severe limitations, experiences symptoms even while at rest). The current state of assistance is a categorical variable with 1 (no external help or intra-familial help only), 2 (assisted living and/or home care services) and 3 (short or long-term care or temporal hospitalization). *ASB* access site bleeding, *NASB* non-access site bleeding, *VASC* vascular complication, *AKIN* acute kidney injury

### Overall, post-discharge and distance-to-death effects during the study period

**Fig. 1:**
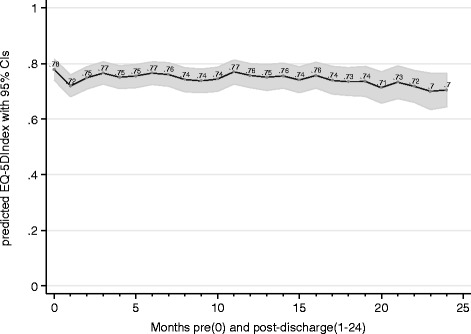
Predicted EQ-5D_Index_ values over the study period

In Fig. 1, the results of a linear regression analysis with a random intercept at the patient level and observational time (in moths) as a categorical covariate are shown.

Over the two-year study period, three main effects were observed. Firstly, QoL measures decreased slightly over time. Secondly, QoL measures were substantially impaired at month 1 after the initial episode of hospitalization. In addition, 39 cases of mortality were reported during follow-up. Of the 39 cases of mortality, 20 % died during the first month post discharge, 33 % died during the first three months post discharge, 49 % died during the first six months post discharge, 78 % died during the first year post discharge and 90 % died during the first 18 months post discharge. QoL decreased substantially at the end of life with a measurable amount starting at the sixth from last follow-up (month) before death.^1^ Table 2 summarizes these three effects on EQ-5D_Index_ values and also shows to which degree the five EQ-5D dimensions (model (2) to (6)) and measures of heart function (model (7) and (8)) are affected by these effects. At the last follow-up before death, for instance, EQ-5D_Index_ values are most impaired by an average of −0.495 (*p* < 0.001) irrespective of time- and post-discharge effects. The same is true for all 5 EQ-5D dimensions (see model (2) to (6) in Table 2) as well as measures of heart function (see model (7) and (8) in Table 2) and the current state of assistance (see model (9) in Table 2). In addition, the current state of assistance is target to a monthly trend, meaning that the risk for worsening of the status of assistance increases by every month of the two year study period (OR 1.06, *p* < 0.05). The same is true for the EQ-5D dimensions of mobility and pain as well as the heart function measured by the NYHA Functional Classification. At month 1 after the initial episode of hospitalization, nearly all QoL measures were substantially impaired, which is also true for the NYHA Functional Classification (OR1.94, *p* < 0.01) and the current status of assistance (OR 2.65, *p* < 0.01). Although both consist of four categories, the NYHA functional classification seems to be more sensitive to trend, post-discharge and time-to-death effects than the CCS grading scale.

**Table 2:** Development of patient HRQOL over time and by distance to death

	Quality of life						Heart function		Status of assistance
	EQ-5D_Index_	Five EQ-5D dimensions							
	(1)	(2)	(3)	(4)	(5)	(6)	(7)	(8)	(9)
	EQ-5D_Index_	Mobility	Looking after myself	Doing usual activities	Having pain or discomfort	Feeling worried, sad or unhappy	NYHA score	CCS score	Status of assistance
	Point estimate	Odds ratios from the ordered logistic regression model							
Last follow-up before death	−0.495^***^	85.56^***^	210.2^***^	79.70^**^	23.66^***^	44.06^***^	4.653	1.258	189.7^***^
Second last follow-up before death	−0.305^***^	14.40^**^	26.95^***^	7.132^*^	16.94^***^	15.65^***^	2.042	2.203	18.08^*^
Third from last follow-up before death	−0.221^***^	11.96	23.42^***^	6.753	2.672	3.567	5.171^**^	1.477	58.83^***^
Fourth from last follow-up before death	−0.204^***^	8.480^*^	10.11^**^	6.338	3.433	8.167^**^	1.807	1.911	22.58^***^
Fifth from last follow-up before death	−0.139^**^	5.282	12.73^**^	8.027	1.384	2.331	1.169	1.247	21.37^*^
Sixth from last follow-up before death	−0.0983^*^	2.033	3.203	2.902	2.918	3.273	1.464	0.996	7.387
Overall trend	−0.00143	1.083^***^	1.040	0.963	1.042^***^	0.979	1.053^***^	1.024	1.060^*^
Month 1 post-discharge	−0.0460^**^	2.878^***^	2.358^*^	2.273^**^	1.391	1.487	1.939^**^	1.197	2.652^*^
Constant	0.789^***^								
*N*	2294	2328	2332	2330	2330	2317	2252	2254	1980

^*^ *p* < 0.05, ^**^ *p* < 0.01, ^***^ *p* < 0.001

The EQ-5D_Index_ is a continuous variable between 0 and 1 (0 = worst state, 1 = best state). The 5 EQ-5D dimensions as shown in Model (2) to model (6) are order categorical variables (1 = no problems, 2 = some problems, 3 = extreme problems). Canadian Cardiovascular Society (CCS) Angina Grading Scale is an order categorical variable between 1 (angina only during strenuous or prolonged physical activity) and 4 (inability to perform any activity without angina). New York Heart Association (NYHA) Functional Classification is an order categorical variable between 1 (cardiac disease, but no symptoms and no limitation in ordinary physical activity) and 4 (severe limitations, experiences symptoms even while at rest). The current state of assistance is an order categorical variable with 1 (no external help or intra-familial help only), 2 (assisted living and/or home care services) and 3 (short or long-term care or temporal hospitalization)

### Between-group differences and baseline-adjustment over the two-year period

According to the European Society of Cardiology/European Association for Cardio-Thoracic Surgery (ESC/EACTS) Guidelines [17], TAVR is recommended for patients considered unsuitable for conventional surgery because of severe comorbidities. In patients considered to be at high risk for conventional surgery, these comorbidities and the associated individual patient’s risk should be assessed by a “heart team” of cardiac surgeons and cardiologists to select the most optimal treatment strategy for individual patients [17]. As a result of this modus operandi, treatment decision is associated with systematic risk selection and the logistic EuroSCORE has previously been shown to be of relevance for predicting both treatment decisions and overall survival [13]. Interestingly, when predicting follow-up QoL measures rather than survival, baseline values of the respective QoL measure had a more dominant impact on future values of any of the observed measures than risk profiles in comparison to the logistic EuroSCORE (see Table 3, where values of the dependent variables at baseline and EuroSCORE values at baseline were included as time-invariant variables). Interestingly, the impact of baseline values on the respective dependent variable seems to persist over the entire two-year period.^2^

**Table 3:** Between-group differences and baseline adjustment when analyzing QoL measures among patients with AS

	Quality of life						Heart function		Status of assistance
	EQ-5D_Index_	five EQ-5D dimensions							
	(1)	(2)	(3)	(4)	(5)	(6)	(7)	(8)	(9)
	EQ-5D_Index_	Mobiliy	Looking after myself	Doing usual activities	Having pain or discomfort	Feeling worried, sad or unhappy	NYHA score	CCS score	Status of assistance
	Point estimate	Odds ratios from the ordered logistic regression model							
Value at baseline	0.321^***^	7.314^***^	18.30^***^	9.356^***^	2.222^***^	2.756^***^	2.419^***^	1.378^***^	9.049^**^
EuroSCORE at baseline	−0.000761	1.029	1.046	1.040^*^	0.999	1.011	0.992	1.000	0.985
TAVR	Reference	Reference	Reference	Reference	Reference	Reference	Reference	Reference	Reference
SAVR	0.0532	0.209^**^	0.754	0.396^*^	0.459^**^	0.413^*^	1.258	0.912	0.0441^***^
SAVRplus	−0.112^*^	3.162	6.843	3.559	2.185	2.487	2.416	1.132	1.129
DRUG	−0.180^**^	14.42^***^	1.463	1.948	2.114	3.667^***^	26.69^***^	1.416	2.174
Last follow-up before death	−0.536^***^	111.5^***^	291.0^***^	149.0^***^	34.40^***^	53.33^***^	4.141	1.816	123.2^**^
Second last follow-up before death	−0.334^***^	16.37^**^	31.55^***^	8.390^*^	19.85^***^	16.00^***^	1.826	1.752	16.09^*^
Third from last follow-up before death	−0.246^***^	15.48^*^	30.99^***^	9.799^*^	3.929	3.782	5.026^*^	1.817	55.18^**^
Fourth from last follow-up before death	−0.225^***^	9.108^*^	12.66^***^	7.110	4.175	8.558^**^	1.670	2.307	24.06^**^
Fifth from last follow-up before death	−0.157^***^	6.018	15.68^**^	11.47^*^	1.822	2.491	0.985	1.371	16.51^*^
Sixth from last follow-up before death	−0.108^**^	2.088	3.711	3.231	3.290	3.457	1.360	1.129	16.89
Overall trend	−0.00140	1.087^***^	1.041	0.967	1.043^***^	0.978	1.051^***^	1.021	1.071^*^
Month 1 post-discharge	−0.0425^**^	2.139^*^	2.038^*^	2.282^**^	1.212	1.346	1.853^*^	1.047	3.206^*^
Constant	0.554^***^								
*N*	2159	2189	2193	2191	2191	2156	2119	1952	1317

^*^ *p* < 0.05, ^**^ *p* < 0.01, ^***^ *p* < 0.001

The EQ-5D_Index_ is a continuous variable between 0 and 1 (0 = worst state, 1 = best state). The 5 EQ-5D dimensions as shown in Model (2) to model (6) are order categorical variables (1 = no problems, 2 = some problems, 3 = extreme problems). Canadian Cardiovascular Society (CCS) Angina Grading Scale is an order categorical variable between 1 (angina only during strenuous or prolonged physical activity) and 4 (inability to perform any activity without angina). New York Heart Association (NYHA) Functional Classification is an order categorical variable between 1 (cardiac disease, but no symptoms and no limitation in ordinary physical activity) and 4 (severe limitations, experiences symptoms even while at rest). The current state of assistance is an order categorical variable with 1 (no external help or intra-familial help only), 2 (assisted living and/or home care services) and 3 (short or long-term care or temporal hospitalization). The group SAVRplus includes 17 patients that underwent additional bypass grafting and/or mitral valve replacement

Furthermore, a number of between-group differences may be observed. In comparison to TAVR patients, SAVR patients report more favorable QoL measures (see model (1) to (6) in Table 3), and, (or maybe because of this), a reduced risk for worsening of the status of assistance is recorded for them (OR 0.0441, *p* < 0.001). On the other hand, an increased risk for limitations in the heart function is recorded for the few patients in the Drug group (see model (7) and (8) in Table 3). Please note that these between-group differences in QoL measures should not, or at least only in part, be interpreted as treatment effects, as the different treatment groups are not randomly assigned but subject to a risk-driven patient selection which may not be fully addressed by the applied baseline-adjustment.

### Clinical events and complications

As shown in Table 1, a number of clinical events were recorded for TAVR and SAVR-patients, of which nearly 80 % occurred during the initial episode of hospitalization. In order to analyze the impact of such events in the short and medium term, Drug patients were excluded from the analyses. As shown in model (1) in Table 4, QoL is most affected by life threatening non-access site bleeding (one-month change: −0.447, *p* < 0.001), stroke (one-month change: −0.161, *p* < 0.05) and stage 3 acute kidney injury (one-month change: −0.177, *p* < 0.01). Interestingly, only life threatening non-access site bleeding was associated with a more intense status of assistance. In a next step, we analyzed the medium term impact of the respective complications. Therefore, categorizations of variables included in Table 5 were restructured by pooling complications of minor interest in ‘other complications’. Then, lagged variables were included in order to capture the medium term impact of the respective complications. As shown in Table 5 model (2), time lags of up to three months were included in the analyses^3^. Interestingly, the impact of lagged effects on EQ-5D_Index_ values remained significant for life threatening access site bleeding only. Other complications, such as life threatening non-access site bleeding (non-ASB), stroke, and stage 2 and stage 3 acute kidney injury (AKIN 2 and 3), are still associated with substantial effects impairments of EQ-5D_Index_ values at the first follow-up interview after event, but the event-related impairments decrease dramatically at the second and third follow-up interviews after event. In Table 5 we also consider the impact of two different strategies to take the influence of the distance to death into account: We may just exclude measurements from the last six months prior to death (models (3) and (4)) or we may just ignore the distance in the modelling (models (5) and (6)) and equal EQ-5D_Index_ to 0 in the month of death. We can observe similar results for 3 complication groups, but changes for non-ASB and AKIN 3. For non-ASB this seems to be related to the fact that this complication took place in 12 patients, of which two died within three months after event. These two patients were facing the worst possible health state EQ-5D_Index_ = −0.205 during each follow-up interview after event and before death. Unsurprisingly, the estimated impact of life threatening access side bleeding changed substantially, as these two patients were excluded from the analysis in model (3) and (4). Basically, the results of model (1) to (4) represent the estimated impact of the respective complication on EQ-5D_Index_ values assuming that the patient survives the six months following the event. Model (5) and (6), in contrast, show the estimated impact of the respective complication on EQ-5D_Index_ values for all patients, including those patients who die shortly after the event. That is why, for instance, model (6) returns substantially higher QoL impairments for patients with AKIN than model (2): Model (2) separates prior-to-death impairments from those arising in patients that do not die during the next months, whereas model (6) does not adjust for upcoming death. Of the sixteen patients facing stage 3 AKIN, nine patients died before the first follow-up after and two patients died during month two and three after event.

**Table 4:** Impact of clinical events on QoL measures among TAVR and SAVR patients

	Quality of life						Heart function		Status of assistance
	EQ-5D_Index_	Five EQ-5D dimensions							
	(1)	(2)	(3)	(4)	(5)	(6)	(7)	(8)	(9)
	EQ-5D_Index_	Mobility	Looking after myself	Doing usual activities	Having pain or discomfort	Feeling worried, sad or unhappy	NYHA score	CCS score	Status of assistance
	Point estimate	Odds ratios from the ordered logistic regression model							
Value at baseline	0.350^***^	14.19^***^	34.09^***^	10.96^***^	1.671^*^	2.751^***^	2.457^***^	1.398^***^	10.82^**^
ASB life threatening/disabling	−0.0463	12.68^**^	0.680	0.199	2.860	0.771	0.123	0.188	0.104
ASB major/minor	0.0985^*^	0.587	0.499	0.201^**^	0.182^*^	0.121^*^	0.762	0.673	0.342
NASB life threatening/disabling	−0.447^***^	584.6^**^	54.93	7.752	0.739	47.86^*^	1.410	1.591	86.32^*^
NASB major/minor	−0.0199	1.188	0.688	0.597	1.311	0.573	1.180	0.813	4.088
VASC major/minor	−0.00695	0.282	0.971	2.029	0.833	2.110	1.422	0.660	6.198
Stroke/TIA	−0.161^*^	8.298^**^	1.358	4.179	6.661	2.561	0.343	0.380	0.958
AKIN 1	0.0659	1.270	1.104	0.357	0.430	0.742	0.547	0.601	0.718
AKIN 2	−0.158^*^	1.297	7.126	102.2^**^	0.814	0.340	81.36^*^	104.2^**^	21.79
AKIN 3	−0.177^**^	15.75^***^	21.53^**^	7.419^*^	2.908	3.495	1.243	1.404	1.955
Arrhythmia	−0.0377	2.393	3.153	1.954	1.817	2.200	3.490	0.234^*^	1.270
Other complications	0.0330	2.617	0.933	0.457	0.366	0.402	1.380	5.237^*^	1.057
Last follow-up before death	−0.493^***^	81.93^***^	154.9^***^	48.14^**^	29.90^***^	32.98^***^	2.877	1.335	144.8^**^
Second last follow-up before death	−0.270^***^	11.50^**^	23.04^***^	5.949	15.13^***^	11.13^***^	1.542	1.786	12.95
Third from last follow-up before death	−0.204^***^	8.142	17.64^**^	4.509	1.996	3.602	3.855^*^	1.377	66.29^**^
Fourth from last follow-up before death	−0.176^***^	7.797^*^	11.09^**^	5.952	2.471	7.750^**^	1.581	2.016	23.22^*^
Fifth from last follow-up before death	−0.150^***^	4.843	13.36^**^	8.091	1.600	2.446	0.926	1.217	25.23^*^
Sixth from last follow-up before death	−0.104^*^	1.800	3.205	2.907	3.803	2.932	1.259	1.081	35.30^*^
Overall trend	−0.00165	1.085^***^	1.045	0.966	1.044^***^	0.983	1.056^***^	1.020	1.088^**^
Month 1 post-discharge	−0.0233	1.602	1.433	2.066^*^	1.465	1.130	1.370	1.599	2.774
Constant	0.528^***^								
*N*	2212	2246	2256	2254	2254	2219	2182	2022	1357

^*^ *p* < 0.05, ^**^ *p* < 0.01, ^***^ *p* < 0.001

The EQ-5D_Index_ is a continuous variable between 0 and 1 (0 = worst state, 1 = best state). The 5 EQ-5D dimensions as shown in Model (2) to model (6) are order categorical variables (1 = no problems, 2 = some problems, 3 = extreme problems). Canadian Cardiovascular Society (CCS) Angina Grading Scale is an order categorical variable between 1 (angina only during strenuous or prolonged physical activity) and 4 (inability to perform any activity without angina). New York Heart Association (NYHA) Functional Classification is an order categorical variable between 1 (cardiac disease, but no symptoms and no limitation in ordinary physical activity) and 4 (severe limitations, experiences symptoms even while at rest). The current state of assistance is an order categorical variable with 1 (no external help or intra-familial help only), 2 (assisted living and/or home care services) and 3 (short or long-term care or temporal hospitalization). *ASB* access site bleeding, *NASB* non-access site bleeding, *VASC* vascular complication, *AKIN* acute kidney injury

**Table 5:** Impact of clinical events on EQ-5D_Index_ among AVR patients accounting for time-to-death

	Time-to-death adjustment (six months)		Exclusion of cases of mortality (six months prior to death)		EQ-5D_Index_ = 0 in month of death	
	(1)	(2)	(3)	(4)	(5)	(6)
	EQ-5D_Index_	EQ-5D_Index_	EQ-5D_Index_	EQ-5D_Index_	EQ-5D_Index_	EQ-5D_Index_
Value at baseline	0.348^***^	0.346^***^	0.333^***^	0.331^***^	0.333^***^	0.331^***^
ASB life threatening/disabling	−0.0593	−0.140^*^	0.0356	−0.0497	−0.0599	−0.121
…at the second follow-up after event		−0.145^*^		−0.155^*^		−0.0993
…at the third follow-up after event		−0.163^**^		−0.0950		−0.145^*^
NASB life threatening/disabling	−0.449^***^	−0.493^***^	−0.457^***^	−0.508^***^	−0.448^***^	−0.465^***^
…at the second follow-up after event		−0.114		−0.140		−0.101
…at the third follow-up after event		0.103		0.0936		0.109
Stroke / TIA	−0.147^*^	−0.206^**^	−0.145^*^	−0.210^**^	−0.147^*^	−0.199^*^
…at the second follow-up after event		−0.123		−0.146^*^		−0.121
…at the third follow-up after event		−0.0319		−0.0286		−0.0307
AKIN 2	−0.167^*^	−0.226^**^	−0.267^**^	−0.270^**^	−0.169^*^	−0.272^**^
…at the second follow-up after event		−0.142		−0.0428		−0.195^*^
…at the third follow-up after event		0.0657		0.0150		0.0508
AKIN 3	−0.173^**^	−0.186^**^	−0.235^**^	−0.293^***^	−0.381^***^	−0.418^***^
…at the second follow-up after event		−0.113		−0.177^*^		−0.235^**^
…at the third follow-up after event		−0.00684		−0.0338		−0.0939
Other complications	0.00548	0.00571	0.0134	0.0140	0.00464	0.00368
Last follow-up before death	−0.493^***^	−0.487^***^				
Second last follow-up before death	−0.275^***^	−0.272^***^				
Third from last follow-up before death	−0.210^***^	−0.217^***^				
Fourth from last follow-up before death	−0.180^***^	−0.189^***^				
Fifth from last follow-up before death	−0.154^***^	−0.166^***^				
Sixth from last follow-up before death	−0.108^**^	−0.116^**^				
Overall trend	−0.00161	−0.00222^**^	−0.00195^*^	−0.00249^**^	−0.00322^***^	−0.00386^***^
Month 1 post-discharge	−0.0230	−0.0283	−0.0256	−0.0301	−0.0163	−0.0218
Constant	0.529^***^	0.543^***^	0.548^***^	0.559^***^	0.508^***^	0.523^***^
*N*	2212	2212	2102	2102	2250	2250

^*^ *p* < 0.05, ^**^ *p* < 0.01, ^***^ *p* < 0.001

The EQ-5D_Index_ is a continuous variable between 0 and 1 (0 = worst state, 1 = best state). *ASB* access site bleeding, *NASB* non-Access Site Bleeding, *VASC* vascular complication, *AKIN* acute kidney injury. Please note that we also increased these time lags up to 6 months after complications but found no additional effects

## Discussion

Due to many potential determinants of patient's QoL after treatment of AS, a careful modelling of longitudinal data is necessary. With such a carefully developed model we were able to demonstrate and quantify short term effects -- and nearly only short term effects -- of the clinical complications considered. Most interestingly, our findings demonstrate that distance to death is one of several factors with clear effects on QoL measures in the elderly population. Our analyses demonstrate a measurable impairment of QoL measures beginning at the sixth from last follow-up (month) before death. Irrespective of the inclusion of time-invariant baseline variables, treatment effects and/or complications, the influence of distance to death remained robust to any specification of the regression models. In order to take into account the effect of distance to death when analyzing QoL measures, repeated QoL measurements and a long follow-up period seem necessary, and detailed information on death during follow-up may be seen as essential.

Unfortunately, these requirements are unmet in the majority of studies analyzing QoL measures among patients with aortic valve stenosis [3, 6–10, 12], as a number of reviews on the topic identified many (observational) studies to be subject to survivor bias [7, 8, 10]. When it comes to cost-effectiveness of different treatment options among patients with aortic valve stenosis, however, the inclusion of valid QoL measures is indispensable. Most of the available literature on the topic includes QoL measures of the well-known Placement of Aortic Transcatheter Valves (PARTNER) trial [3, 18–29]. The PARTNER trial takes a special place among the literature due to its large patient population and its status as the first and for a long period only randomized multicenter study of QoL outcomes under medical care, TF-TAVR, TA-TAVR, and SAVR so far. Sehatzadeh et al. [11] summarize the body of literature on the medical and QoL results of this trial, as well as the incremental cost-effectiveness ratios of the various techniques, at two years of follow-up.

Strikingly, within the boundaries of the study design and inclusion/exclusion criteria, there appear to be a number of important differences in patients from either the TAVR or the other groups [13]. Therefore, it may be misleading to make direct comparisons of the results between the groups. Moreover, our initial study design included the enrollment of drug patients, analogous to the conservative treatment arm from PARTNER B [30], but it soon became evident that these patients were rarely considered inoperable and were typically offered TAVR. This directly reflects the current clinical practice and demonstrates the very limited number of patients presenting with a “true” contraindication for TAVR or SAVR. However, it remains unclear whether these “borderline” patients are a consequence of a shift in perceptions about eligibility for TAVR or surgery, and if so what impact their inclusion will have on mortality and morbidity affecting comparisons with pivotal studies such as PARTNER [3, 20, 30].

Please note that there are a number of limitations: First of all, between-group differences in QoL measures should not, or at least only in part, be interpreted as treatment effects, as the different treatment groups are not randomly assigned but subject to a risk-driven patient selection which may not be fully addressed by the applied baseline adjustment. Secondly, there are substantial decreases in QoL measurement compliance over the two year period and we may not assure whether dropouts were entirely noninformative.

## Conclusion

Finally, cost-effectiveness analyses highly depend on valid information regarding the impact of relevant clinical events. Interestingly, our results indicate that in the present dataset of elderly patients, only few complications had a sustained long-term impact on QoL. Again, the appropriate inclusion of death seems crucial in terms of model selection for estimating the impact of clinical complications on QoL values.

## Abbreviations

AKIN, acute kidney injury; AS, aortic valve stenosis; CCS, canadian cardiovascular society; EACTS, european association for cardio-thoracic surgery; ESC, european society of cardiology; HRQOL, health-related quality of life; non-ASB, non-access site bleeding; NYHA, New York heart association; QoL, quality of life; SAVR, surgical aortic valve replacement; TAVR, transcatheter aortic valve replacement; TCCT, TAVI calculation of costs trial; VARC, valve academic research consortium

## Footnotes

1 Neither the extension of the time period before death to seven or more follow-up months before death, nor the extension of the post-discharge effect to two or more months showed a measurable impact on QoL measures and was therefore excluded from the regression models.

2 The inclusion of interaction terms between EQ-5D_Index_ values at baseline and time showed no substantial differences regarding their impact on EQ-5D_Index_ values at month 1, during year 1 post-discharge, or later than one year post-discharge.

3 Extension of the lagged effect did not show an impact on QoL measures and was therefore excluded from the regression models.
